# The "Romsås in Motion" community intervention: program exposure and psychosocial mediated relationships to change in stages of change in physical activity

**DOI:** 10.1186/1479-5868-4-15

**Published:** 2007-04-30

**Authors:** Catherine Lorentzen, Yngvar Ommundsen, Anne Karen Jenum, Ingar Holme

**Affiliations:** 1 Norwegian School of Sport Sciences, P.O. BOX 4014, Ullevål Stadion, 0806 Oslo, Norway; 2 Aker Diabetes Research Centre, Faculty Division Aker University Hospital, 0514 Oslo, Norway

## Abstract

**Background:**

Conducting process evaluations of health promoting interventions, and measuring the effectiveness of specific intervention components, may help in the understanding of program failure or success. The purposes of the present study were to examine adults' exposure to and involvement in specific components of a three year long pseudo-experimental community-based physical activity intervention, and to examine the relationship between such exposure and participation and changes in stages of change in physical activity and psychosocial mediators.

**Methods:**

1497 persons in the intervention group attended the baseline survey in 2000 (50.6%) and 1204 (80.4 of baseline attendees) provided data on the outcome variables of the present study. In 2003, 1089 were still living in the area, and were re-invited to follow-up assessments. Current analyses are based on the 603 persons (mean age 49 ± 10 years) who provided baseline and follow-up data for the current purposes (56.6% follow-up rate). Process data, stages of change in physical activity, and potential psychosocial mediators of change in physical activity were assessed by questionnaires. The theory-based intervention was composed of communication, physical activity, environmental and participatory components. Data were analysed using frequency and descriptive statistics, Chi-square and t-tests, and regression analyses.

**Results:**

Exposure and participation rates in the various intervention components varied greatly (1.5–92.7%). Participation in walking groups and aerobic exercise groups, as well as having seen the "Walk the stairs"-poster were significantly and positively related to change in stages of change in physical activity (β = .12, p = .011; β = .211, p < .001; β = .105, p = .014, respectively). Additionally, having used the walk path was significantly and positively related to change in stages in women (β = .209, p = .001) but not in men (β = -.011, p = .879), and in Western people (β = .149, p = .003) but not in non-Westerners (β = -.293, p = .092). Observed significant relations were partly mediated by positive changes in psychosocial factors as social support from friends, perceived control, and physical activity identity.

**Conclusion:**

Findings revealed that particular intervention components, such as participation in physical activity groups, were more strongly related to forward transition in stages of change in physical activity than others. These findings together with results indicating that such transitions were mediated by specific psychosocial influences may improve theory and help to prioritize among specific intervention components in future programs.

## Background

Previous interventions promoting physical activity have shown mixed results [1], and little is known about why some were successful and others not [2]. Conducting process evaluation of intervention implementations may help to increase such an understanding [3,4]. For example, data from a cardiovascular disease prevention program revealed that the poor intervention effects on physical activity and its determinants might be attributed to the relatively small fraction (up to 15 %) of the population being reached by intervention strategies [5]. However, previous large scale integrated programs are typically evaluated as whole projects, which makes it difficult to isolate the effect of particular elements of the intervention [2]. Assessing data concerning exposure to specific intervention components would enable such analyses [2,6].

Recently, examination of mediators of physical activity intervention effects [7], and of the relationship between the quantity of implementation of the intervention, mediators and outcomes [8], have been requested. The construct of stages of change in physical activity may be useful as a behavioural outcome measure in physical activity promoting interventions [9]. Testing psychosocial mediators of movement in the stages of change [10-12] resulting from exposure to or participation in different intervention components [10,13] may provide important information about how specific intervention components work. This may improve intervention theory, physical activity behaviour change methods, strategies and intervention tools [2,6]. Previous research has shown that the most promising psychosocial mediators of movement in stages of change in physical activity include self-efficacy beliefs, social support [14,15], attitude, perceived behavioural control [16], and identity [17].

Finally, subgroup analyses should be performed as for instance gender, age and ethnicity may be moderators of the attractiveness of different intervention strategies, or of the impact of participating in a specific intervention component on stages of change in physical activity or psychosocial variables [6]. For example, the possibility for social interaction in group activities may make such intervention components more attractive for women than for men [18]. Such information may be helpful in designing interventions specifically for subgroups of a population [6].

We have previously reported the positive results of a pseudo-experimental multiple-component community-based physical activity intervention aimed at reducing the burden of type 2 diabetes and cardiovascular disease in an adult population [19]. After three years of intervention, the proportion reporting to be physically inactive was reduced by 25%, weight gain was 50% of that in the control community, and beneficial changes in other risk factors for type 2 diabetes and cardiovascular disease were also observed. The main purposes of the present study were to examine a) peoples' exposure to and participation in specific intervention components, b) whether such exposure and participation were related to change in stages of change in physical activity, and c) whether any such relationships were mediated by psychosocial influences and moderated by socio-demographic and anthropometric factors.

## Methods

### Design and procedure

The data from the current study were drawn from the "Romsås in Motion" project. The project design, baseline results and main outcomes have been reported elsewhere [19,20] (Lorentzen, Ommundsen, Jenum, & Holme, submitted). Pre- and post- intervention surveys in two suburban communities in Oslo in year 2000 and 2003 included a physical examination comprising height and weight measurements, the collection of venous blood samples as well as a main questionnaire (Q1) assessing information on health status and health-related behaviours. In addition, a specially designed questionnaire concerning physical activity (Q2), comprising a measure of the stages of change in physical activity [21] and several measures of theory-based possible psychosocial mediators of change in physical activity [22-24], was handed out at the survey site and returned by mail. All instruments underwent translation and back-translation procedures, and were pilot-tested on a small sample. At the post-intervention survey, participants in the intervention district also completed a third questionnaire (Q3), also pilot-tested on a small sample, assessing exposure to and participation in specific intervention components [3]. Questionnaire data and Body Mass Index (BMI) were used in current analyses. Participants provided their written informed consent at both surveys. The Regional Ethics Committee and The Norwegian Data Inspectorate approved the study protocol.

### Participants

All individuals between 31 and 67 years in the intervention community were invited to participate in the baseline health survey (n = 2954). Of these, 1497 attended (50.7%), and 1204 (80.4% of the attendees) provided data on the outcome variables from Q2 (stages of change in physical activity and psychosocial influences). Based on registry information from Statistics Norway of the invited cohort, the attendees to the baseline survey had a slightly higher socio-economic status than the non-attendees [20]. Furthermore, the proportion of participants of non-Western origin who provided baseline outcome data (16.4%) was lower than for those not attending the baseline survey (26.6%, p < .001) or providing these data.

Of the attendees, 1089 were alive and still living in the Oslo area in 2003, and were re-invited to follow-up assessments. Of these, 755 (69.3%) attended and 616 (56.6%) provided follow-up data on outcome variables from Q2. Of these, 603 answered at least one question from Q3, and were included in the analyses of the present study, representing an attrition rate from baseline of 44.6%.

### Intervention

**Figure 1:**
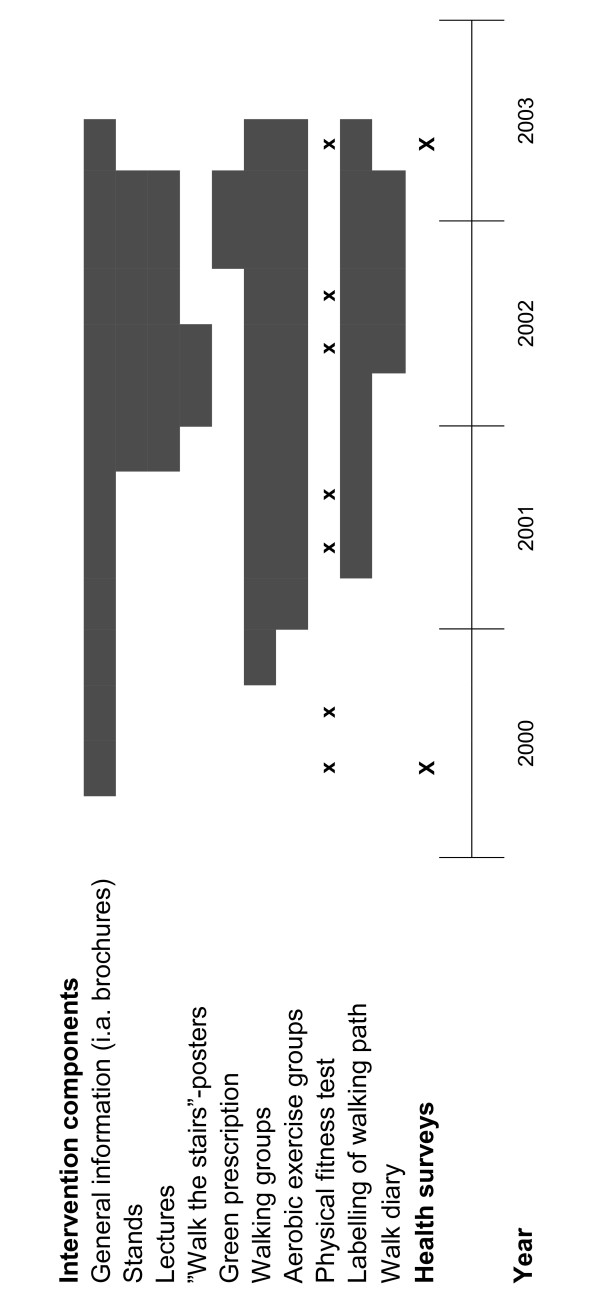
Intervention components and time of exposure in relation to the health surveys.

The theory-based intervention aimed at generating a forward transition in stages of change in physical activity by favourably influencing the potential psychosocial mediators; social support, self-efficacy, attitudes, perceived control, and physical activity identity. The multi-component population-based intervention (Figure 1) comprised four main strategies:

#### 1) Communication strategies

A range of communication efforts were developed and implemented throughout the intervention period to: 1) communicate information about physical activity and health, and to 2) promote the physical activity programs of the project [25]. A number of channels and settings were employed; local television, radio, newspapers, posters, stage-based and ordinary brochures, direct mailings, stands, lectures etc. "Walk the stairs"- posters [1] were placed at block entrances and in public buildings to encourage people to climb the stairs in stead of using escalators and elevators. Late in the intervention period, the local General Practitioners also prescribed physical activity (Green prescription). As about 20% of the target population were of non-Western origin [20], efforts were made to reduce language and cultural barriers by translating some of the information material into the most common foreign languages and by offering a special program to the attendants at Norwegian classes.

#### 2) Physical activity programs

Low-threshold physical activity programs were developed in close collaboration with representatives of the inhabitants of the community [3]. The activities comprised a number of weekly sessions of outdoor walking groups and indoor aerobic exercise programs conducted by exercise leaders, a dance course, as well as a test of physical fitness twice a year.

#### 3) Environmental strategies

In accordance with social-ecological models [12] several initiatives were implemented to increase the accessibility to physical activity arenas in the local environment; labelling of walking paths and improved street lighting, snow clearing and gritting of pavements and walking paths during winter season. To increase the motivation to use the walking paths, a walk diary was distributed to every household in the community, and could be returned to project staff after registration of weekly walking distance for participation in local competitions.

#### 4) Participatory strategies

The intervention was based on participatory approaches [26], including local political and lay leaders as well as local health and welfare workers in the planning and implementation of intervention strategies. Furthermore, the project was incorporated in the strategic plans of the community, stating the councils' commitment to and involvement in the project.

The multiple strategy approach was designed to reach individuals at different stages of readiness to adopt and maintain physical activity. In general, communication strategies emphasizing physical activity and health aimed at changing perceptions, knowledge, and attitudes towards physical activity and thereby enhancing motivational readiness for physical activity among individuals in the first stages of change, while the physical activity programs and the environmental strategies targeted people already motivated to increase their physical activity [27]. The exposure time for specific components varied throughout the three-year intervention (see Figure 1). A gradual and orchestrated introduction of components was planned to facilitate synergetic effects of each component on peoples' psychological readiness and behaviour change. Several intervention components were, however, due to resource limitations, introduced later than planned. In addition to these population-based intervention strategies, individuals with type 2 diabetes or cardiovascular diseases, or having high risk for these diseases at the baseline medical examination (n = 76), were offered medical follow-up and individual and group counselling on physical activity, dietary habits and smoking during the intervention period.

### Measurement

#### Process evaluation

The questions concerning participants' exposure to and participation in specific intervention components included in the process evaluation questionnaire (Q3) are displayed in Table 1.

**Table 1:** Percentage of sample and baseline subgroups reporting being exposed to and participating in specific intervention components.

	Whole sample	Women	Men	< 50 years	≥ 50 years	Western	Non-Western	Low education	High education	Low BMI	High BMI	Inactive	Active
	n = 402– 598	n = 347	n = 256	n = 262	n = 341	n = 550	n = 53	n = 295	n = 303	n = 297	n = 305	n = 221	n = 382
Noticed the project	95.3	95.9	94.5	95.8	95.0	96.7***	81.1	94.8	95.7	93.2*	97.4	91.2***	97.6
Heard about the physical fitness test	92.7	92.9	92.5	94.6	91.2	93.4	86.0	91.3	94.0	92.4	93.0	87.8**	95.6
Participated in the physical fitness test	50.8	49.3	52.7	46.4	54.4	51.3	45.0	44.2**	56.3	51.0	50.8	45.6	53.6
Heard about the walking groups	90.6	92.2	88.3	89.4	91.5	91.9**	78.0	90.1	90.8	88.4	92.6	84.7***	94.0
Participated in walking groups	9.9	10.3	9.4	4.7***	14.7	9.9	10.5	13.5*	6.8	7.7	12.2	11.5	9.0
Heard about aerobic exercise groups	81.4	85.5**	75.5	79.9	82.7	83.3**	63.3	82.2	80.4	80.3	82.8	77.5	83.7
Participated in aerobic exercise groups	13.3	17.9**	6.6	7.6**	18.5	13.8	8.1	15.3	11.3	12.9	13.7	11.0	14.5
Heard about the walk path	80.6	83.1	77.2	78.9	82.0	84.4***	39.5	80.7	80.2	80.2	81.3	74.7*	84.1
Used the walk path	21.7	24.2	18.3	18.4	24.8	22.8	8.8	20.3	22.4	20.4	23.2	13.3**	26.0
Heard about the walk diary	58.0	62.9**	50.7	54.5	61.0	61.3***	24.4	57.8	57.9	60.1	56.2	54.3	60.2
Participated in the walk diary	6.4	6.6	6.2	4.9	7.9	6.5	6.1	5.7	7.1	7.5	5.3	4.2	7.6
Heard about the Green prescription	38.1	41.8*	33.0	36.5	39.5	40.0**	19.0	38.2	37.6	37.6	38.5	37.3	38.6
Received the Green prescription	1.5	1.3	1.7	1.0	2.0	1.4	3.1	1.7	1.4	1.0	2.0	0.0	2.2
Heard about lectures	29.3	29.9	28.5	27.4	31.0	29.4	28.3	31.0	27.6	25.5*	32.9	28.0	30.1
Participated at lectures	3.7	3.8	3.4	3.4	3.9	3.7	2.9	3.8	3.6	1.9*	5.5	2.9	4.0
Seen stands	45.4	43.0	48.7	43.6	46.9	47.2**	27.1	45.2	45.2	44.3	46.7	35.9**	50.9
Seen brochures	75.8	77.6	73.5	79.6	72.9	76.9	64.7	71.5*	79.9	73.7	77.8	66.7***	81.0
Seen "Walk the stairs"-poster	41.7	43.3	39.7	38.5	44.3	42.1	37.5	45.3	38.4	41.6	42.0	37.7	44.0

* = p < .05, ** = p < .01, *** = p < .001. Western = born in Western Europe, North America, Australia or New Zealand. Education low = <12 years; high = ≥12 years. BMI low = <26.3 kg/m^2^; high = ≥26.3 kg/m^2^. Inactive = Precontemplation, Contemplation; active = Preparation, Action, Maintenance.

#### Stages of physical activity behaviour change

Stages of change in physical activity were measured using a question developed by Prochaska and Marcus [21]. The stages were assessed using a scale of five categories labelled as: 1) "I am currently not physically active and I do not intend to engage in physical activity in the next 6 months" (Precontemplation), 2) "I am currently not physically active, but I am thinking about getting more physically active in the next 6 months" (Contemplation), 3) "I currently do some physical activity, but not regularly" (Preparation), 4) "I am currently regularly physically active, but I have only begun doing so within the last 6 months" (Action), and 5) "I am currently regularly physically active and have done so for more than 6 months" (Maintenance). Respondents were asked to think of all physical activity except work-related activity when choosing a response category. This was done because the main target behaviours of the intervention were physical activity from the other domains, as these are considered more modifiable [28]. No definitions of physical activity regularity, intensity and frequency were given. The psychometric properties of the stages of change in physical activity measure have been reported elsewhere (Lorentzen et al, submitted). In some analyses, the stages of change were merged to create a dichotomized inactive-active variable. Persons in the two lowest stages (Precontemplation and Contemplation) were classified as inactive, while persons in the other stages (Preparation, Action, and Maintenance) were classified as active [20].

#### Psychosocial mediators

Measures of psychosocial mediators of physical activity behaviour change were included; social support [29], self-efficacy [30], attitudes [31,32], perceived behavioural control [32], and identity [24,33,34]. More detailed descriptions of these variables and their internal consistency properties are summarized in Table 2. Details on the psychometric construction of these mediators have been reported elsewhere (Lorentzen et al, submitted).

**Table 2:** Summary of psychosocial variable measurements.

Variable	Number of items/response format	Example of sample items	Definition of physical activity	Original reference source on which items were based	Cronbach's alpha, pre- – post-test
*Social support*		How often, over the last three months, did...	None	29	
- family	11/1 (none) -5 (very often)	... your family/friends, acquaintances, co-workers do physical activities with you?			0.92 – 0.92
- friends, acquaintances, and co-workers	11/1 (none) -5 (very often)				0.93 – 0.93
*Self-efficacy*		I am confident I can participate in planned physical activity when...	All physical activity, except work-related physical activity	30	
- psychological barriers	7/1 (not at all confident) -7 (extremely confident)	... I am tired			0.92 – 0.92
- practical barriers	5/1 (not at all confident) -7 (extremely confident)	... I have a lot of work to do			0.79 – 0.73
					
*Attitude*		Being regularly physically active the next month will to me be ...	None	31,32	
- Evaluative	5/1–7	... useless-useful			0.91 – 0.91
- Affective	3/1–7	... unpleasant-pleasant.			0.86 – 0.87
					
*Perceived behavioural control*	4/1–7	How sure are you that you could be regularly physically active the next month? unsure – sure	All types of physical activities	32	0.83 – 0.82
*Identity*	4/1 (suits badly) – 5 (suits well)	To what degree do these statements describe you as a person: I view myself as a person who is concerned about physical activity	None	24,33,34	0.92 – 0.92

#### Socio-demographic and anthropometric variables

Data on gender, age, and country of origin were available for the invited cohort from Statistics Norway. In some analyses age was dichotomized to those aged less than 50 and those aged 50 or more. Participants born in Western Europe, North America, Australia and New Zealand were categorized as Western. Years of education, employment status, and disability pension status were self-reported. BMI was calculated from the participants' weight in kilograms and height in meters squared (kg/m^2^), measured as part of the physical examination. In some analyses education and BMI were dichotomized into low and high categories by their median values (12 years and 26.3 kg/m^2^, respectively), to enable subgroups analyses on groups of comparable size.

### Statistical analyses

Differences in baseline frequency distributions and means by population subgroups were investigated by Chi-square statistics and independent samples t-tests, respectively.

According to Baron and Kenny [13], several steps are required to demonstrate a mediation effect: 1) the intervention affects the outcome, 2) the intervention affects the hypothesized mediator, 3) change in the hypothesized mediator is associated with change in the outcome, and 4) the relationship between the intervention and the outcome is attenuated when controlling for change in the hypothesized mediator. The higher the attenuation of the effect, the greater is the potency of the mediator [35]. As the stages of change in physical activity construct previously had shown linear relationships with several independent psychosocial variables [31,36,37], also in the total intervention study sample (Lorentzen et al., submitted), linear multiple regression analyses were considered appropriate.

To test criteria 1 and 2, change in stages of change in physical activity and changes in psychosocial variables were regressed on variables expressing exposure to and participation in (yes/no) the different intervention components. These analyses were performed separately for each intervention component and each outcome. When both criteria 1 and 2 were met, a third regression analysis was conducted to test criteria 3 and 4, where change in stages of change in physical activity was regressed on both exposure and change in the hypothesized mediator.

To test for moderating effects by gender, age, ethnicity, education and BMI in the relationships between exposure to or participation in intervention components and change in stages of change in physical activity (criteria 1), stepwise hierarchical regression analyses with change score in stages of change in physical activity as the dependent variable were performed. The baseline level of the moderator variable was entered in step 1, exposure to or participation in the intervention component was entered in step 2, and in step 3 the interaction term of exposure to or participation in the intervention component × the baseline moderator variable was entered. This procedure was repeated separately for each of the potential moderators and for each of the intervention components that related significantly to change in stages.

Change scores (post test – pre test) of stages of change in physical activity and of each potential psychosocial mediator were used in the regression analyses. The significance level for all analyses was set at *P *< .05. Although the risk for making a type I error (incorrectly declaring a statistical significance) increases with multiple testing, the significance level was not adjusted for multiple comparisons for reasons outlined by various authors [38,39]. The number of cases available for each analysis varied due to missing data. All analyses were performed with the Statistical Package for the Social Sciences (SPSS) (version 13.0).

## Results

### Sample characteristics

Baseline socio-demographic characteristics, distribution in stages of change in physical activity, and level of psychosocial variables for the sample are presented in Table 3. The majority of participants were of Western origin (91.2%), mean BMI was 27.1 kg/m^2^, more than 60% of the participants reported that they were inactive or irregularly active (Precontemplation, Contemplation, and Preparation stages), and mean values on evaluative and affective attitudes towards physical activity were high. Those who were included in the present study were older (p < .001), had higher education (p < .01), and were more likely to be of Western origin (p < .001) than those lost to follow-up (see Table 3).

**Table 3:** Baseline socio-demographic and physical activity related characteristics of participants by inclusion to study.

	Included*	Not included	p
	n = 603	n = 486	
	M (SD)	M (SD)	
*Socio-demographic and anthropometric characteristics*			
Age (years)	49.4 (9.9)	45.8 (10.3)	.000
Women (%)	57.5	55.8	.555
Non-Western (%)	8.8	27.2	.000
Education (years)	12.0 (3.6)	11.4 (3.8)	.006
Full-time employment (%)	63.1	59.0	.167
Disability pension (%)	18.5	19.3	.756
BMI (kg/m^2^)	27.1 (4.7)	27.0 (5.1)	.712
			
*Stages of physical activity behaviour change (%)*			
Precontemplation	17.2	18.9	
Contemplation	19.4	19.3	
Preparation	25.7	26.5	
Action	5.6	5.3	
Maintenance	32.0	29.8	.918
			
*Psychosocial variables*			
Social support, family	2.0 (0.8)	2.1 (0.9)	.308
Social support, friends	2.0 (0.8)	2.0 (0.8)	.306
Self-efficacy, psychological barriers	4.4 (1.7)	4.4 (1.8)	.774
Self-efficacy, practical barriers	3.6 (1.4)	3.7 (1.4)	.154
Attitude, evaluative	6.2 (1.2)	6.2 (1.2)	.649
Attitude, affective	5.3 (1.5)	5.3 (1.5)	.645
Perceived behavioural control	4.7 (1.6)	4.6 (1.6)	.407
Identity	3.2 (1.2)	3.3 (1.2)	.880

*Providing baseline and follow-up data on outcome variables, and answering the process evaluation questionnaire.

BMI, body mass index.

Social support and Identity range from 1 to 5; Self-efficacy, Attitude, and Perceived behavioural control range from 1 to 7. Higher scores indicate a greater psychosocial readiness for physical activity.

### Exposure and participation rates

95.3% of respondents reported that they had noticed the "Romsås in Motion" project. Exposure to the different intervention components (Figure 1) varied a lot (Table 1). For example, 92.7% had heard about the physical fitness test, 75.8% had seen project brochures about physical activity, and 41.7% had seen the "Walk the stairs"-poster. Actual participation in intervention activities varied considerably, from 50.8% (at least once accomplished the test of physical fitness) to 1.5% (received a Green prescription from their physician).

Subgroup analyses (Table 1) revealed that, compared to their counterparts, a significantly higher proportion of Westerners, people with high BMI, and physically active persons at baseline had noticed the project and had been more exposed to and participated more in some intervention components. The latter also held true for women and persons aged 50 years or more.

### Exposure, participation, and forward transition in stages of change in physical activity

As indicated by the β-weights in Table 4, participation in walking groups and aerobic exercise groups, having used the walk path, and seen the "Walk the stairs"-poster were significantly related to forward transition in stages of change in physical activity. Interaction analyses demonstrated gender (change in R^2 ^of step 3 p < .05) and ethnicity (change in R^2 ^of step 3 p < .01) interaction effects with respect to using the walk path. The relationship between having used the walk path and change in stages of change in physical activity was significant for women (β = .21, p < .01, n = 255) but not for men (β = -.01, p = .88, n = 185), and for Western persons (β = .15, p < .01, n = 407), but not for non-Westerners (β = -.29, p = .09, n = 33).

**Table 4:** Relationship between intervention components exposure, change in stages of physical activity, and psychosocial mediating influences (n = 324–578).

	**A**			**B**		**C**		**D**	
			
**Intervention components**	**β**	**p**	**Potential psychosocial mediators**	**β**	**p**	**β**	**p**	**β**	**p**
Heard about the physical fitness test	-.002	.970	Social support, family	-.105	.029				
			Identity	.103	.021				
Participated in the physical fitness test	.065	.138	Attitude, affective	.109	.020				
			Identity	.141	.002				
Heard about the walking groups	.048	.266							
Participated in walking groups	.120	.011	Social support, friends	.153	.004	.173	.001	.095	.074
			Perceived behavioural control	.109	.025	.358	.000	.091	.046
Heard about aerobic exercise groups	.086	.051	Identity	.136	.003				
Participated in aerobic exercise groups	.211	.000	Social support, family	.177	.001	.235	.000	.164	.001
			Social support, friends	.252	.000	.152	.005	.151	.005
			Self-efficacy, psychological barriers	.148	.003	.284	.000	.155	.001
			Attitude, affective	.117	.021	.231	.000	.154	.002
			Perceived behavioural control	.198	.000	.367	.000	.142	.002
			Identity	.230	.000	.309	.000	.155	.001
Heard about the walk path	.038	.386	Identity	.120	.009				
Used the walk path	.121	.011							
*Women (n = 202–255)*	.209	.001	Social support, friends	.186	.008	.240	.001	.147	.034
			Perceived behavioural control	.223	.000	.476	.000	.102	.075
*Men (n = 150–185)*	-.011	.879	Social support, friends	.206	.011				
			Self-efficacy, psychological barriers	.156	.044				
			Perceived behavioural control	.211	.004				
*Westerners (n = 329–407)*	.149	.003	Social support, friends	.198	.000	.195	.000	.129	.019
			Self-efficacy, psychological barriers	.105	.043	.289	.000	.129	.010
			Perceived behavioural control	.234	.000	.348	.000	.072	.136
*Non-Westerners (n = 33)*	-.293	.092							
Heard about the walk diary	.039	.391							
Participated in the walk diary	.043	.377	Social support, friends	.119	.028				
			Self-efficacy, psychological barriers	.111	.031				
			Perceived behavioural control	.118	.018				
Heard about the Green prescription	.050	.268							
Received the Green prescription	-.038	.451	Social support, friends	.113	.043				
Heard about lectures	.041	.371							
Participated at lectures	.082	.098	Identity	.151	.003				
Seen stands	.056	.190	Social support, friends	.118	.015				
			Perceived behavioural control	.207	.000				
			Identity	.136	.002				
Seen brochures	.073	.080	Perceived behavioural control	.188	.000				
			Identity	.131	.002				
Seen "Walk the stairs"-posters	.105	.014	Social support, friends	.155	.001	.171	.000	.086	.075
			Perceived behavioural control	.182	.000	.390	.000	.041	.321
			Identity	.089	.043	.319	.000	.076	.068

A = Relationship between exposure to or participation in specific intervention components and change in stages of physical activity.

B = Relationship between exposure to or participation in specific intervention components and change in potential psychosocial mediators.

C = Relationship between change in potential mediators and change in stages of physical activity.

D = Relationship between exposure to or participation in specific intervention components and change in stages of physical activity adjusted for change in mediator.

Note. All βs refer to the standardized regression coefficients from simple and multiple regression analyses.

Only significant relationships are included in B. C and D are included when both A and B are significant.

### The role of psychosocial mediators

For all the intervention components which related to forward transition in the stages of change in physical activity, indication of psychosocial mediation was revealed. First, changes in all psychosocial variables related with the actual intervention component (step 2 in the Baron & Kenny formula) related significantly and positively to change in stages of change in physical activity (all p's<.05) (step 3 in the formula). Additionally, the β weights indicating the relationship between exposure to intervention components and change in stages (step 1 in the formula) were reduced when controlling for changes in the actual psychosocial variables (step 4 in the formula). Thus, the positive relation between having participated in walking groups and change in stages of change in physical activity was partly mediated by favourable changes in support from friends (β reduced .025 from step 1 to step 4 in the formula) and perceived control (reduction β = .029). The positive relation between having participated in aerobic exercise groups and change in stages was partly mediated by favourable changes in support from family (reduction β = .047) and from friends (reduction β = .06), self-efficacy faced with psychological barriers (reduction β = .056), affective attitudes (reduction β = .057), perceived control (reduction β = .069), and physical activity identity (reduction β = .056). The positive relation between having seen the "Walk the stairs"-poster and change in stages was partly mediated by favourable changes in support from friends (reduction β = .019), perceived control (reduction β = .064), and identity (reduction β = .029). Due to interaction concerning the walk path, mediation analyses for this specific intervention component were performed only for women and for Westerners. For women, social support from friends and perceived control mediated the observed significant relation between having used the walk path and change in stages of change in physical activity. For Westerners, support from friends, self-efficacy in the face of psychological barriers, and perceived control mediated this relation (see Table 4).

## Discussion

Having participated in walking groups and aerobic exercise groups, having seen the "Walk the stairs"-poster, and having used the walk path (women and Westerners only) were significantly and positively related to change in stages of change in physical activity. Observed significant relations between exposure to and participation in intervention components and forward transition in the stages of change were partly mediated by positive changes in several psychosocial factors. Among the stronger mediators were support from friends, perceived control, and identity.

### Exposure and participation rates

Variations in exposure and participation rates between different intervention components were expected due to the gradual introduction (Figure 1). The higher exposure than participation rates are consistent with the Persuasion Communication model postulating many steps in the process between initial exposure to a communication message and actual change in behaviour [3]. Although differences in intervention programs, their duration and measurement methods make direct comparisons of results difficult, the level of intervention exposure found in our study is comparable to that of another study using a mass-media based multi-component intervention [40], promoting walking among sedentary 50- to 65-year-olds. Many study participants (32–90%) had been exposed to information through various media channels, but less than 5% reported seeing or participating in any of the public health education programs.

The significantly higher proportion of women compared to men who reported having participated in aerobic exercise groups is in accordance with other studies [18], although we did not find gender differences concerning participation in other intervention group activities. Not surprisingly, the non-Westerners were more difficult to reach with information messages than Westerners, despite the efforts made to reduce language and cultural barriers. Although exposure and participation rates in most intervention components were comparable for people with different educational status and BMI level, the higher participation rates of persons with low education in walking groups and of persons with high BMI at lectures are encouraging as these groups are considered hard to reach in health promoting work [1]. Overall, high proportions of initially inactive persons were reached by intervention components, although less than among those who were physically active at baseline. This is in line with previous findings suggesting that present physical activity behaviour is related positively to past physical activity behaviour [24].

### Exposure, participation, and forward transition in stages of change in physical activity

Findings suggest that having participated in walking and in aerobic exercise groups, having used the walk path, and having seen the "Walk the stairs"-poster were the most effective in moving intervention participants forward towards a higher stage of change. The sizes of the beta values suggest that taking part in aerobic exercise groups may have contributed the most. Our results are comparable with previous research. For example, several reviews have found support for the effectiveness of point of choice prompts to encourage stair use rather than escalators or lifts [1,2]. Results concerning the walking groups fit nicely with the current emphasis on walking within public health interventions. Indeed, due to its acceptability and accessibility, walking groups seem particularly attractive among populations with low levels of physical activity [41].

Having used the walk path seemed to favourably influence stage movement in women but not in men, and in people of Western origin, but not in non-Westerners. The walk path passes by apartment buildings, a shopping centre, and public transportation terminals. Hence, the result may reflect that women and Western inhabitants used this path more in the purpose of exercising, and that men and non-Western people to a higher degree used the path as a means of transportation for accomplishing everyday tasks, which may not have influenced their level of motivation for participation in physical activity or their perceived levels of physical activity. No other moderating effects were found. Apparently, participating in walking and aerobic exercise groups, or seeing a "Walk the stair"-poster, seems equally able to influence stage movement, irrespective of gender, age, ethnicity, education level or BMI group.

### The role of psychosocial mediators

The results of this study correspond well with and complement previous mediation analyses on the same material, indicating that several of the same psychosocial factors acted as mediators of the favourable intervention effect on stage movement (Lorentzen et al., submitted). The findings suggest that specific intervention components may have triggered specific psychosocial factors to influence the forward stage transition (Lorentzen et al., submitted). For instance, participating in aerobic exercise groups seems to play an important role in the previously observed increase in perceived support from family in the intervention group (Lorentzen et al., submitted). The latter is in line with former research emphasizing the great opportunity of gaining social support for physical activity behaviour by attending exercise groups [42].

As expected, all intervention components that significantly related to change in stages of change in physical activity seemed to partly have worked through increasing participants' general perceived control over physical activity behaviour [22,32]. Enhancement of peoples' perception of "low-treshold" physical activity opportunities in the local environment may have increased their perceived control over physical activity. This line of reasoning fits well with socio-ecological models stating that the environment may promote and maintain health-enhancing physical activity [43].

Several factors may explain why not all intervention components influenced potential psychosocial mediators of physical activity behaviour change or stage movement. First, a couple of these components were implemented rather late in the intervention period (Figure 1). Second, the low participation rates in some of the components resulted in low power for these groups in statistical analyses, making it difficult to obtain significant relationships. Indeed, similar intervention strategies have been found effective in increasing physical activity in previous research. For instance, comparable with the Green prescription, several studies have demonstrated that primary care physicians may play a significant role in the promotion of physical activity [44]. Hence, more research is needed to rule out the potential effect of these components.

### Strengths and limitations

The main strengths of the present study include the measurement of indications of effect of specific intervention components on change in stages of change in physical activity [2], combined with the examination of numerous theory-derived potential psychosocial mediators of such changes [7]. Second, the investigation of potential moderating factors in these analyses also provides important information [6].

Inherent in the present study are also several methodological limitations. Foremost, process evaluation data were retrospective and self-reported, increasing the possibility for both recall bias and social desirability bias [45]. For instance, people who have changed their behaviour after being exposed to specific intervention components may be more likely to remember the components than people who did not change their behaviour. Recall and social desirability bias may also apply to the self-reported stages of change in physical activity and psychosocial factors. For example, Ronda and colleagues [46] reported that more than 60% of those insufficiently active overestimated their activity level. The results of the current study may also be limited by the lack of a comparison condition. The non-controlled, retrospective evaluation design reduces the ability to make certain conclusions about the effects of the specific intervention components on psychosocial influences and stages of change. However, several of the psychosocial variables positively related to exposure to and participation in different intervention components were the same as those found to be positively affected by the whole intervention strategy package in analyses applying a prospective controlled design (Lorentzen et al., submitted). This, together with the significant favourable intervention effect on forward stage transition revealed in the same study, support the main findings of the present study.

For better understanding the effects of the present intervention, more detailed process evaluation information, such as the reading of brochures, the receipt of information at stands, or reasons for participating or not in intervention components, could have been assessed [3]. Low response rates at baseline (40.8 %) and at follow-up (55.4 %) may also have biased the results. As attrition analyses indicated that baseline and follow-up samples comprised people with slightly higher education and more Westerners compared to those not attending or not providing relevant data at these assessment time-points, the results may not be generalized to the same extent to these subgroups. Further, since successful promotion of physical activity is recognized as more challenging in groups of low socio-economic status and groups of ethnic minorities [1], the results observed in the present study might have been less positive if such selection effects had not taken place. However, no differences were found on baseline values of other socio-demographic characteristics, BMI, distribution in stages of change in physical activity, or psychosocial mediators between those who completed both assessments and those lost to follow-up, making the present sample fairly representative of those providing baseline data. These findings also indicate that those who provided data for the present study did not have a higher baseline psychological readiness for increases in physical activity than those who did not. However, people who have become more physically active as a result of the intervention might still be overrepresented, and the present sample was characterized by a relatively high baseline physical inactivity rate and BMI score, which further limits generalization of results.

The results should also be interpreted with caution as some say that the stages of change construct should not be used as a proxy for physical activity change, as movement between some stages do not involve change in physical activity level [47]. However, the significant positive relationship found between the stages of change in physical activity and strenuous physical activity in leisure and commute time in the main study (Lorentzen et al., submitted) lends support for the use of the stages of change construct as an outcome measure. Finally, the lack of a precise definition of regular physical activity may have reduced the reliability and validity of the stages of change measure, as Martin-Diener and colleagues [48] found that an individual can be in different stages of change depending on the definition of the behaviour.

## Conclusion

The present study indicated that women, people aged 50 years or more, persons of Western origin, and persons with high BMI were the most easily reached by the actual intervention approach. Participation in walking groups and aerobic exercise groups, using the walk path, and seeing the "Walk the stairs"-poster seemed the most effective intervention components in terms of moving people forward in the stages of change in physical activity. Such changes may partly have occurred through the improvement of psychosocial factors such as support from friends, the extent to which performance of physical activity behaviour is perceived as easy or difficult, and the extent to which one identifies oneself as a physically active person. These results may be used to inform researchers and intervention planners about which specific elements to include in future interventions.

## Competing interests

The author(s) declare that they have no competing interests.

## Authors' contributions

CL participated in the collection of data and in the implementation of the intervention, analysed the data and drafted the manuscript. AKJ participated in the implementation of the intervention. YO, AKJ, and IH participated in the design of the overall study project, and provided critical revision of the paper and its analyses. All authors read and approved the final manuscript.
